# Multiple epigenetic modification profiles reveal the tumor immune microenvironment and clinical outcomes of uveal melanoma

**DOI:** 10.3389/fgene.2023.1155199

**Published:** 2023-04-12

**Authors:** Xinshuai Nan, Yuchen Liu, Yuzhen Gao, Xinshan Nan

**Affiliations:** ^1^ Department of Ophthalmology, Subei Peoples’ Hospital Affiliated to Yangzhou University, Yangzhou, China; ^2^ Department of Ophthalmology, Yangzhou Aier New Vision Eye Hospital, Yangzhou, China; ^3^ Department of Clinical Laboratory, Sir Run Run Shaw Hospital, Zhejiang University School of Medicine, Hangzhou, China; ^4^ Department of ICU, Hua Xin Hospital, Ningbo, China

**Keywords:** uveal melanoma, methylation, tumor microenvironment, immune features, prognostic signature

## Abstract

Uveal melanoma (UM) is an aggressive intraocular cancer that, in 50% of cases, spreads to the patient’s other systems. The exact cause of the increased metastatic rate is still unknown. Methylation and immune response, metastasis, and the expansion of cancer cells are closely related. Additionally, proteins linked to RNA methylation have come to light as possible cancer treatment targets. However, the relationship between methylation-related genes (MRGs) and the tumor microenvironment (TME) is still not understood. The goal of this work was to discover important MRGs and create a signature for UM patients’ prognosis prediction. Using two different data sets, we examined the MRG expression patterns in the transcriptional and genomic regions of 106 UM samples. We discovered a connection between the clinicopathological traits of the patients, their prognosis, the capability of TME cells to infiltrate, and various MRG changes. Following that, we developed an MRGs signature to forecast prognosis, and we evaluated the model’s precision in patients with UM. We grouped the patients into multiple categories based on their clinical traits, looked at the survival rates for various groups within various groupings, and tested their accuracy. Additionally, to increase the practical usability of the MRGs model, we created a very accurate nomogram. TIDE scores were higher in the low-risk group. We go over how MGRs could impact UM’s TME, immunotherapy responsiveness, prognosis, and clinically significant features. We looked for different chemotherapeutic drugs and cutting-edge targeted agents for patients in diverse subgroups in order to better understand MRGs in UM. This helped in the creation of customized therapy to open new doors. We could also further research the prognosis and develop more efficient immunotherapy regimens.

## 1 Introduction

UM is the most common primary intraocular tumor in adults and the most common non-skin kind of melanoma, with a wide variation in its incidence from 0.1 to 8.6 per million by age, ethnicity, and latitude (Rantala et al., 2022). UM develops from melanocytes in the uveal canal, most frequently in the choroid, unlike cutaneous melanoma (CM), and it has different genomic alterations and molecular profiles than the more prevalent CM (Spagnolo et al., 2012). Despite efficient means of removing the original tumor, such as enucleation or less frequently, local radiotherapy. Metastatic illness, which affects 50% of patients and develops regardless of initial ocular treatment, is now untreatable (Bustamante et al., 2021). Sadly, metastatic UM has a high death rate within 6–12 months (Killock, 2021).

It is well known that RNA methylation and the associated downstream signaling cascades have an impact on a variety of biological processes, including sex determination, stress response, cell differentiation, and others (Yang et al., 2018). The most prevalent alteration in most eukaryotic mRNAs, N6-methyladenosine (m^6^A), participates in nearly all phases of the RNA life cycle, including translation, destruction and RNA transcription (Ma et al., 2019). In eukaryotes, there is a significant abundance of the post-transcriptional alteration known as N1-methyladenosin (m^1^A) (Motorin and Helm, 2022). Additionally, recent research has demonstrated that m^1^A alterations can control mRNA translation. Five-methylcytosine (m^5^C) can control ribosome biogenesis, regulate translation when it appears on tRNA or rRNA, and influence the stability and translation of mRNA when it exists on mRNA (Sergiev et al., 2018; Motorin and Helm, 2022). N7-methylguanosine (m^7^G) is one of the most prevalent base alterations in post-transcriptional control. It plays a crucial role in regulating RNA processing, metabolism, stability, nucleation, and protein translation and is abundantly distributed in the 5'cap region of tRNA, rRNA, and eukaryotic mRNAs (Dai et al., 2021). Methylation-binding proteins read it, demethylases (FTO and ALKBH5) demethylate it, and RNA methyltransferases (YTHDF1 and IGF2BP1) catalyze the process (Motorin and Helm, 2022). It is important in the formation and development of a large number of immune system diseases, such as cancer and a wide range of other human pathogenic activities (Papanicolau-Sengos and Aldape, 2022). The m^6^A/m^1^A/m^5^C/m^7^G alteration has been found to contribute to cancer initiation, advance malignancy, and promote recurrence, in addition to playing a significant role in the pathogenesis of a number of human diseases, including immunological disease and neurological disorders (Dawson and Kouzarides, 2012; Ferrier and Burnier, 2020; Wang et al., 2022). Although RNA methylation is clearly important in various malignancies, nothing is known about the connection between m^6^A/m^1^A/m^5^C/m^7^G-associated genes and UM (Robertson et al., 2017; Chokhachi Baradaran et al., 2020; Ferrier and Burnier, 2020).

RNA methylation fluctuations in cancer have been identified as prospective candidates for the creation of diagnostic, prognostic, and predictive biomarkers. However, it is unclear how certain methylation regulators may affect the prognosis and conceivable biological causes of UM (Robertson et al., 2017). Jing Tang et al. state that m^6^A RNA methylation regulators are one of the recently identified biomarkers for the potential malignant progression and prognostic value of UM and may be regarded as a new promising biomarker for the development of UM prognosis and treatment approaches (Tang et al., 2020). Significant variations in the methylation of several genes, including NFIA, HDAC4, and IL12RB2, were also observed in UM, according to research by Ferrier and Burnier (2020). Role of Epigenetics in UM by Yongyun Li et al. summarized that numerous epigenetic changes, such as variations in the expression levels of miRNA, hypermethylation of tumor suppressor genes, histone modification patterns, and hypomethylation of oncogenes are clearly related to the development of UM tumors and many other cancers (Li et al., 2017). Despite the fact that methylation is important for both carcinogenesis and anticancer pathways, very little research has addressed its significance in tumors, particularly in UM (Spagnolo et al., 2012). Very little research has looked at the possibility that the altered methylation pattern may be a factor in the metastatic phenotype. As a result, greater research into the precise methylation modifications found in these tumors is required to better understand the factors that affect UM prognosis and identify potential novel treatment targets. Given the promise that epigenetic-targeted medications have shown in many tumor types, either through targeting particular changes directly or through targeting epigenetic regulators, the correction of epigenetic aberrations may be a potential strategy for preventing metastasis in UM (Farooqi et al., 2019; Miranda Furtado et al., 2019; Ilango et al., 2020). Monitoring the precise changes in UM methylation that are linked to a greater risk of metastasis would also reveal how the tumor reacts to various therapy options. Given the high rate of metastasis in UM and its dismal prognosis, this is especially significant.

For UM patients and other hard-to-treat cancer forms, immunotherapies show promise as successful therapies. They have revolutionized the field of cancer treatment. Clinical studies for immunotherapies, including checkpoint inhibitors, vaccinations, and T-cell treatments, are being conducted on an increasing number of UM patients (Orloff, 2021). However, a significant portion of patients had little to no therapeutic effect, which falls woefully short of meeting a clinical need (Rossi et al., 2021). Multiple studies have revealed that the tumor microenvironment (TME) also has a significant impact on the cancer’s growth (Roma-Rodrigues et al., 2019; Jin et al., 2021; Martínez-Reyes and Chandel, 2021). Cancer cells were able to escape hypoxia, promote growth, decrease apoptosis and angiogenesis, and develop immunological tolerance through interactions with some TME components (direct and indirect) (Deepak et al., 2020). As we become more aware of the diversity and complexity of the microenvironment that tumors depend on, emerging research demonstrates that it has an important place in tumor growth, immunotherapy response, and immune escape (Bejarano et al., 2021). The fact that the response to ICB was anticipated in accordance with the features of TME cell infiltration is a critical step in maximizing the efficiency of currently available ICBs and applying cutting-edge immunotherapeutic techniques (Bejarano et al., 2021; Marseglia et al., 2021). A study revealed a significant relationship between the MRG score for UM and immune infiltration (Jia et al., 2019). According to accumulating evidence, different types of T cells are essential elements of the immunological defense against UM (Fu et al., 2022). Cancer-infiltrating T cell concentrations in UM samples were higher than those in healthy tissues, indicating a better prognosis (Jin et al., 2021). In order to discover different cancer immune phenotypes and improve the ability to predict and guide immunotherapeutic responsiveness, the complexity and variety of the TME landscape should be carefully analyzed. The quest for new therapeutic targets will be aided by the identification of very accurate biomarkers that will assess patients’ responses to immunotherapy.

In recent years, bioinformatics technology has continued to develop, and multi-omics technologies such as genomics, transcriptomics, and proteomics have gradually become the key to facilitate proper treatment of clinical diseases (Olivier et al., 2019; Guo et al., 2022). In the research, we thoroughly assessed the expression of methylation regulators in 79 UM samples from The Cancer Genome Atlas (TCGA) dataset as well as the correlation of genetic alterations with clinical traits and validation in 27 UM samples from the Gene Expression Omnibus (GEO) dataset. We examine both the general promoter methylation pattern and specific loci that are highly differentially methylated depending on the patient’s risk level to demonstrate the importance of specific methylation modifications in UM on cancer progression. The information is crucial in identifying potential targets for a more accurate prognosis and treatment of this lethal eye cancer.

## 2 Materials and methods

### 2.1 Preprocessing of data

The TCGA-UM and GEO-GSE84976 databases provided the RNA-seq data and clinical details for UM (van Essen et al., 2016). 79 UM samples were included in the TCGA-UM dataset, while 27 UM samples were included in GSE84976. From earlier research, we gathered 88 methylation-related genes (MRG) (Li et al., 2022; Shao et al., 2022; Wu et al., 2022) (Supplementary Table S1). We used the limma package in the R program to evaluate DEGs.

### 2.2 Development and verification of model

Prognostic MRGs were identified using univariate Cox analysis (*p* < 0.01), and a risk model was created using multivariate Cox analysis. Each UM patient’s risk score was calculated using an algorithm: $\sum_{i=1}^{k}\beta iSi$ . To validate this model, the GEO-GSE84976 dataset was used as an external validation set. To compare the survival rates of different groups, a Kaplan-Meier analysis was used. To assess the accuracy of survival prediction, receiver operating characteristic (ROC) curves and the area under curve (AUC) were used.

Based on clinical characteristics, we divided the patients into several categories and investigated the survival rates for different groups within various groupings. The model was tested using univariate and multivariate Cox analyses to ensure that it was an accurate predictor of prognosis. The consistency index (C-index) was used to calculate the model’s accuracy. A nomogram was developed to forecast the 1, 3, and 5-year survival rates of UM patients using the model and clinical data.

### 2.3 Enrichment and mutation frequency analysis

The Gene Ontology (GO) and Kyoto Encyclopedia of Genes and Genomes (KEGG) enrichment analysis was performed on the differentially expressed genes (DEGs) between different groups (|logFC > 2| and FDR 0.05) (Wu T. et al., 2021). The number of gene mutations was determined by the use of mutational analysis.

### 2.4 Assessment of the tumor immune microenvironment landscape

To calculate differences in immune cell infiltration and immunological function, we used a single-sample Gene Set Enrichment Analysis (ssGSEA). To examine the levels of several immune checkpoint genes’ expression, the Wilcoxon signed rank was used. To forecast immunotherapy response, the tumor immune dysfunction and exclusion (TIDE) scores were calculated (Fu et al., 2020).

### 2.5 Recognition of anti-tumor drugs

To assess the anti-tumor medications utilized in the clinical treatment of UM, we calculated the half inhibitory concentration (IC50) of medicines using the “pRRophetic” R package and compared the IC50 between different groups (Bakhoum et al., 2021).

## 3 Result

### 3.1 Development and verification of risk assessment signature

**FIGURE 1:**
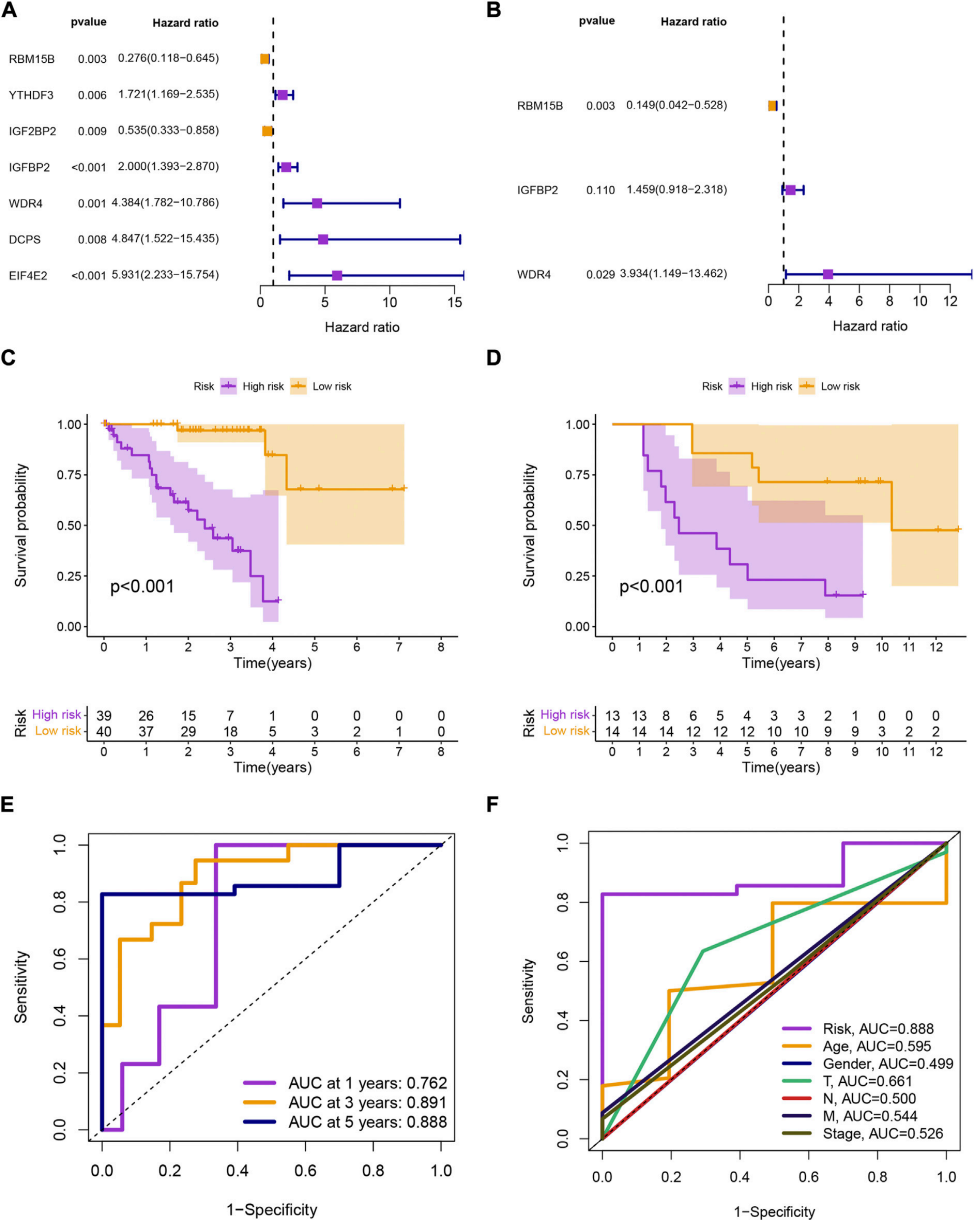
**(A**, **B)** Univariate and multivariate Cox analyses of overall survival for uveal melanoma patients based on clinical characteristics and gene expression signature. **(C**, **D)** Kaplan-Meier survival analysis of uveal melanoma patients in TCGA-UM and GSE84976 datasets stratified by high and low risk scores based on the gene expression signature. **(E)** Area under the curve (AUC) values of the receiver operating characteristic (ROC) curves for the gene expression signature and other clinical features. **(F)** AUC comparison of the gene expression signature with other clinical features.

Univariate Cox analysis identified 7 prognostic MRGs (*p* < 0.01; Figure 1A), and multivariate Cox analysis created a signature with 3 prognostic MRGs (Figure 1B). The high-risk group had a shorter survival time (*p* < 0.001; Figure 1C), and the validation set from GSE84976 had identical results (*p* < 0.001; Figure 1D). The signature was used to forecast UM patients’ 1-, 3-, and 5-year survival rates, with the corresponding AUC values of 0.762, 0.891, and 0.888 (Figure 1E). The model’s AUC was higher than that of other clinical characteristics, demonstrating its greater reliability (Figure 1F).

**FIGURE 2:**
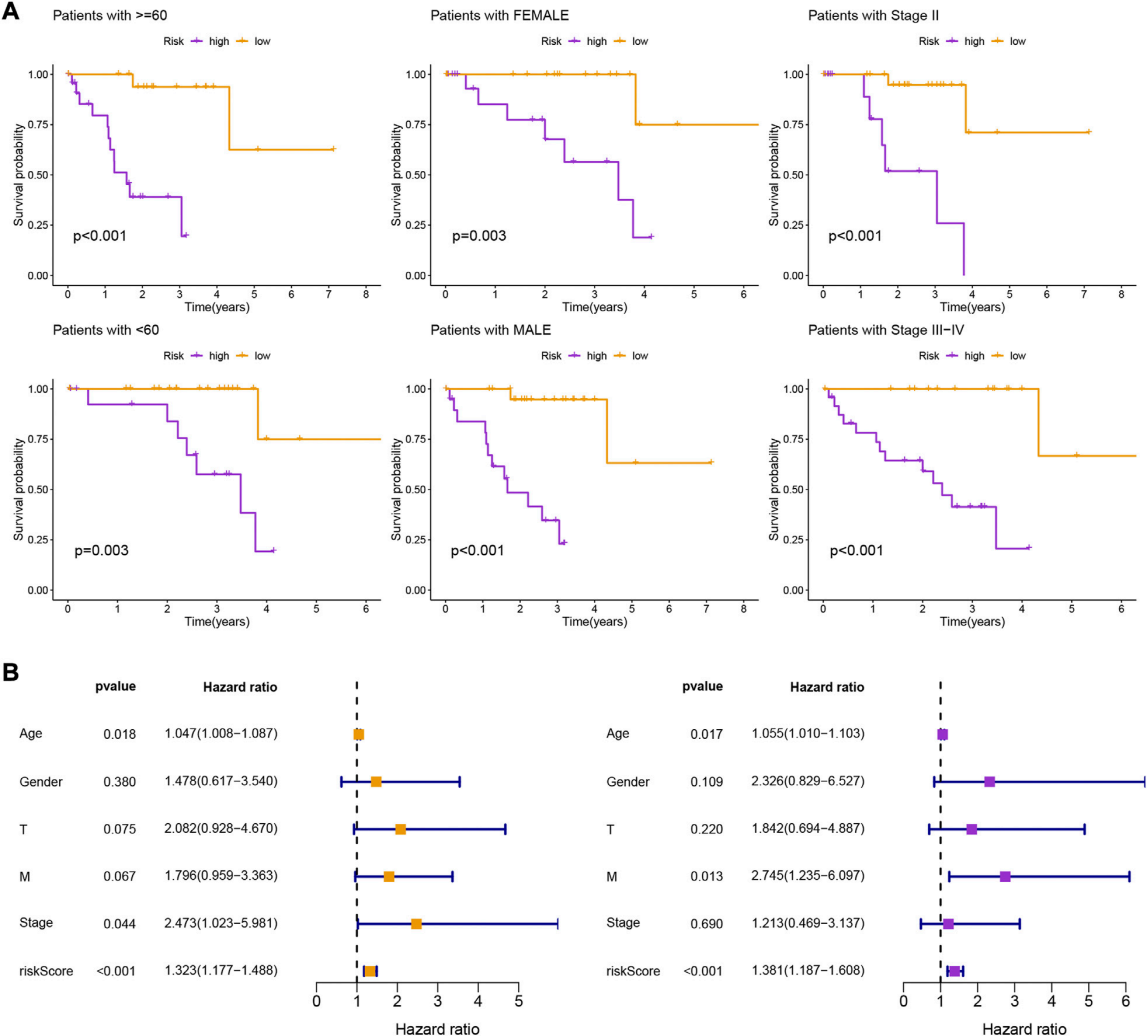
**(A)** Kaplan-Meier survival curves of uveal melanoma patients in different clinical groupings (I-IV) based on high and low risk scores derived from the gene expression signature. **(B)** Kaplan-Meier survival curves of uveal melanoma patients stratified by high and low risk scores regardless of other clinical factors, showing that the risk score is a robust prognostic factor that can be used to stratify patients into different risk groups.

**FIGURE 3:**
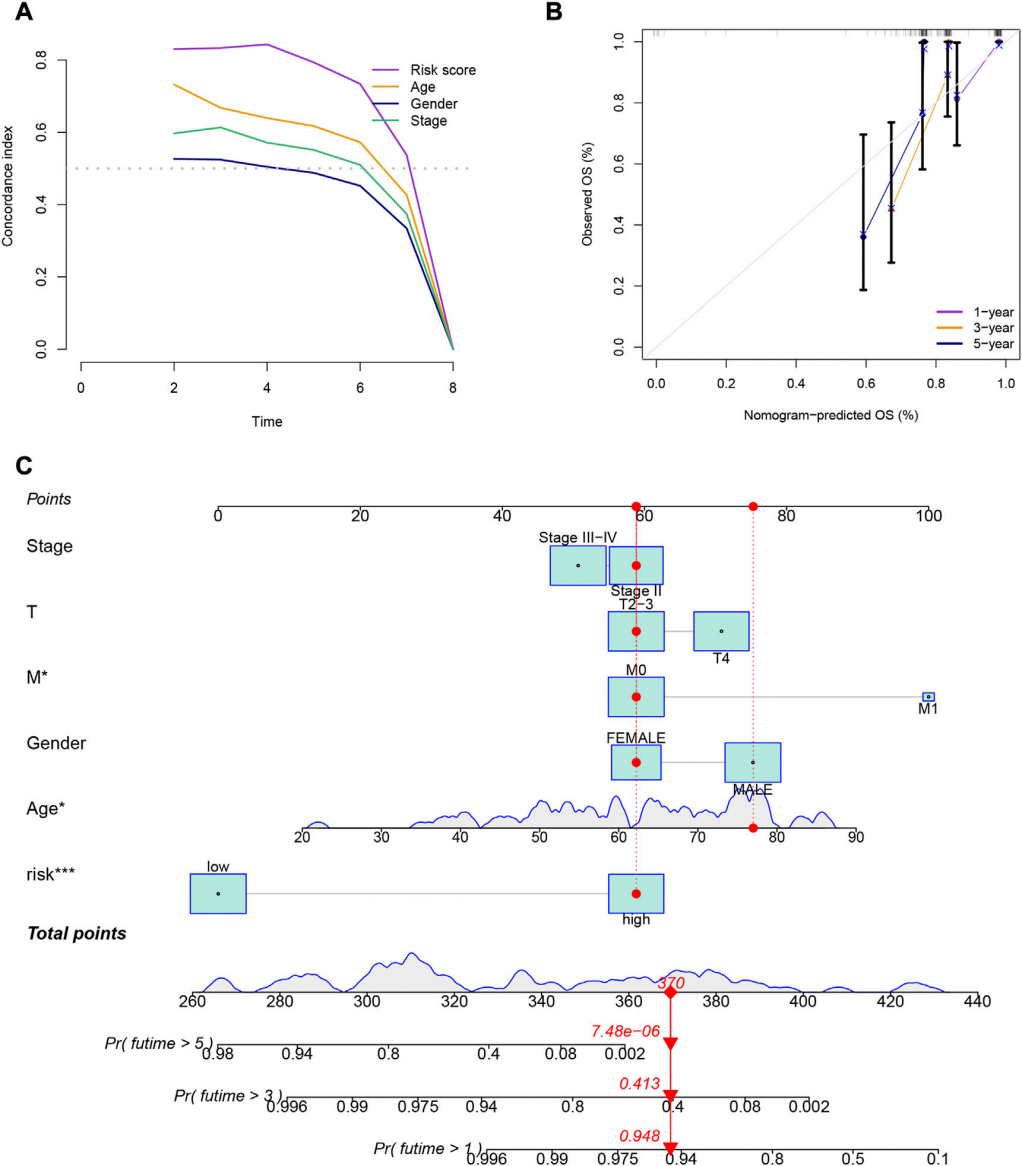
**(A)** Comparison of the gene expression signature with traditional clinical characteristics in predicting the prognosis of uveal melanoma patients. **(B)** Correlation plot of the predicted and observed survival rates at 1, 3, and 5 years. **(C)** Nomogram incorporating the gene expression signature and clinical characteristics to predict the prognosis of uveal melanoma patients.

Patients in the low-risk group had higher survival rates, based on the various clinical subgroups, suggesting that the model is applicable to patients with a range of clinical features (Figure 2A). In both univariate and multivariate Cox analyses, the risk score was shown to be an independent prognostic factor (*p* < 0.001; Figure 2B). The C-index showed that the model performed better at predicting the prognosis for UM than did traditional clinical criteria (Figure 3A). The correlation plot showed that the observed 1, 3, and 5-year survival rates and the predicted rates strongly agreed (Figure 3B). We developed a nomogram containing the signature and clinical characteristics that might be used to precisely predict UM patient survival (Figure 3C).

### 3.2 Enrichment and mutation frequency analysis

**FIGURE 4:**
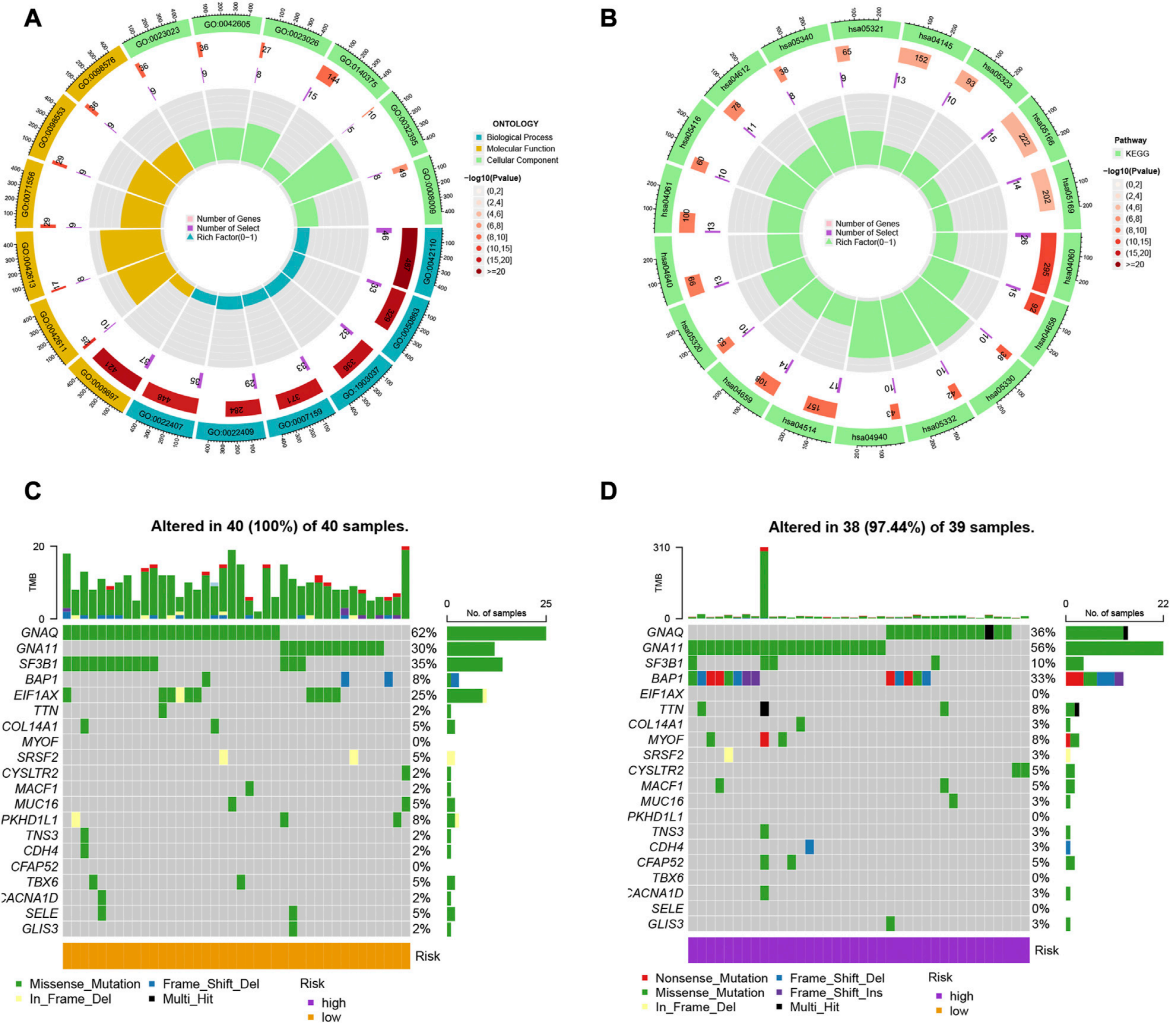
**(A**, **B)** Gene Ontology (GO) and Kyoto Encyclopedia of Genes and Genomes (KEGG) pathway analyses of the differentially expressed genes (DEGs) in uveal melanoma. **(C**, **D)** Comparison of the prevalence and specific altered genes of gene mutations across different risk groups.

We identified 314 DEGs between different risk groups to investigate the various molecular pathways (Supplementary Table S2). Figures 4A, B show the results of the GO and KEGG analyses, while Supplementary Tables S3, S4 give more information. Although the prevalence of gene mutations was comparable across groups, the specific altered genes varied (Figures 4C, D). Different genetic mutations can lead to different outcomes.

### 3.3 Assessment of immunological landscape

**FIGURE 5:**
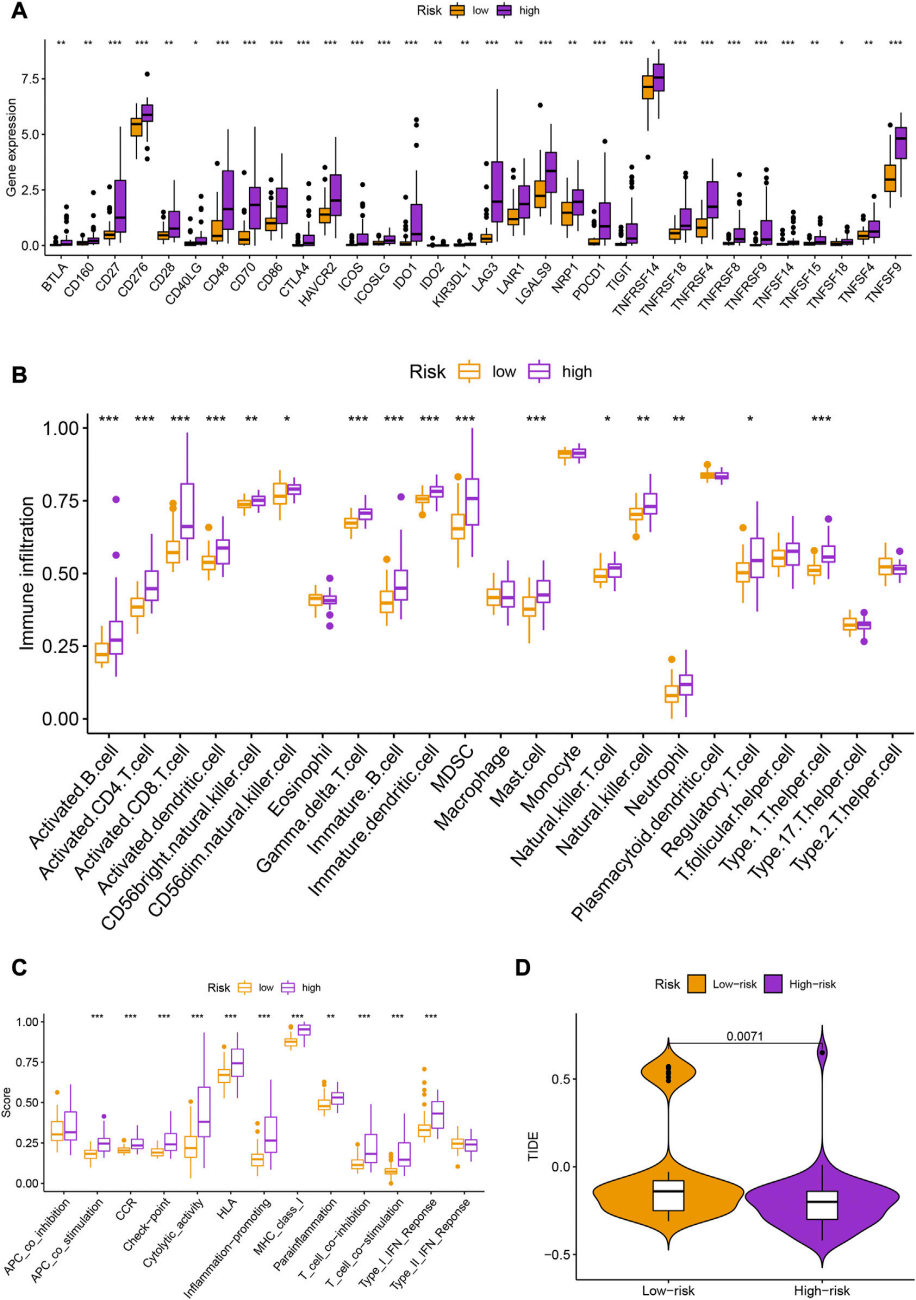
**(A)** Box plots showing the expression levels of immune checkpoint genes (CTLA-4, PDCD1, LAG3, TIGIT, and IDO1) in different risk groups. **(B)** Heatmap showing the differences in immune cell infiltration across different risk groups. **(C)** Enrichment scores of different immunological processes in different risk groups. **(D)** Tumor Immune Dysfunction and Exclusion (TIDE) scores for different risk groups.

There were also statistical differences between risk groups in the expression of genes linked to immune checkpoints, such as CTLA-4 (*p* < 0.001), PDCD1 (*p* < 0.001), LAG3 (*p* < 0.001), TIGIT (*p* < 0.001), and IDO1 (*p* < 0.001) (Figure 5A). Other immune cell infiltrations were substantially different across groups in addition to eosinophils, macrophages, monocytes, plasmacytoid dendritic cells, T follicular helper cells, type 17 T helper cells, and type 2 T helper cells (Figure 5B). Other immunological processes apart from APC co-inhibition and type II IFN response differed considerably between groups (Figure 5C). The high-risk group had lower TIDE scores (*p* = 0.0071; Figure 5D), indicating that they would probably respond to immunotherapy better.

### 3.4 Selection of anti-tumor drugs

**FIGURE 6:**
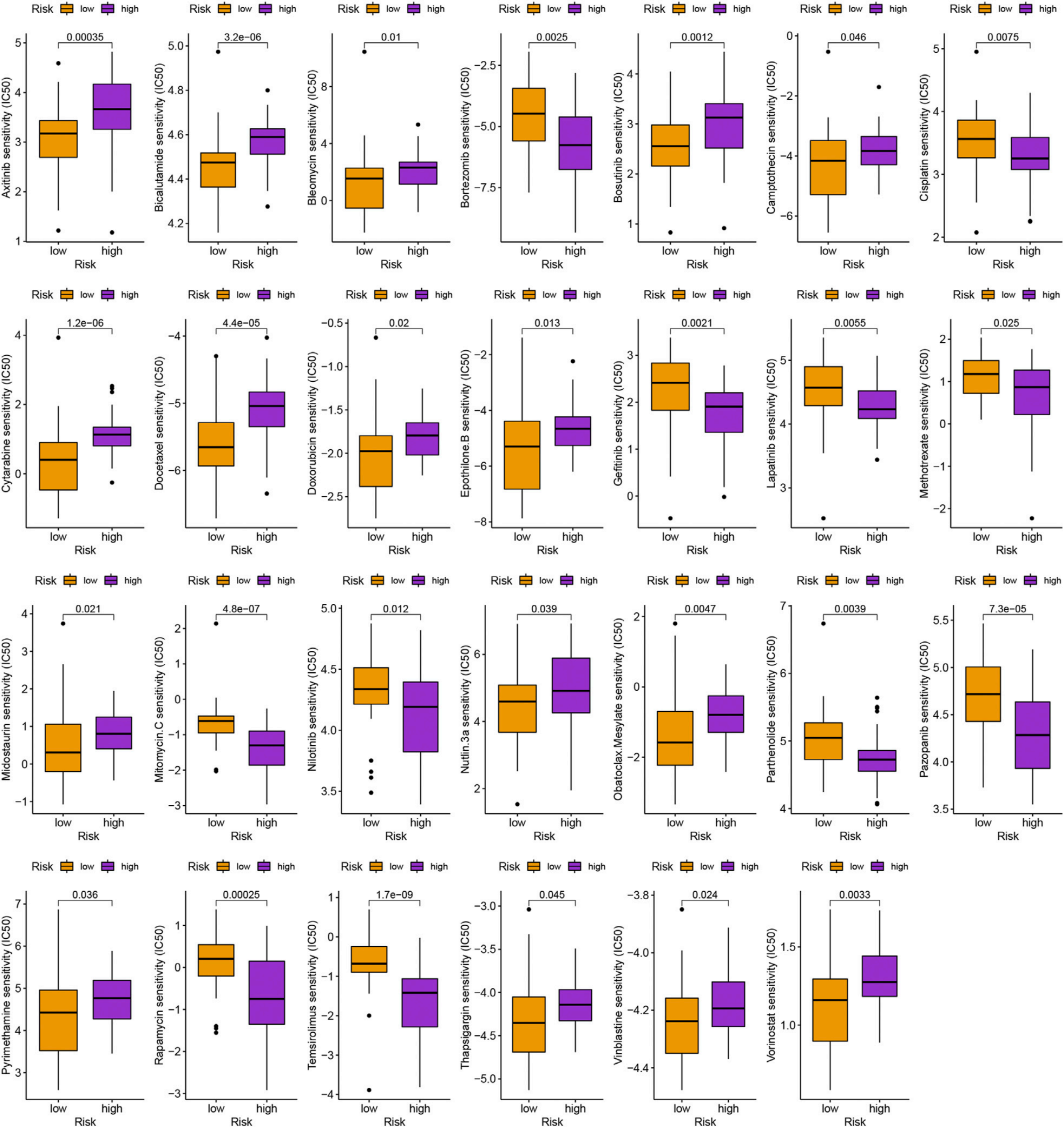
Schematic representation of conventional chemotherapy agents for uveal melanoma.

**FIGURE 7:**
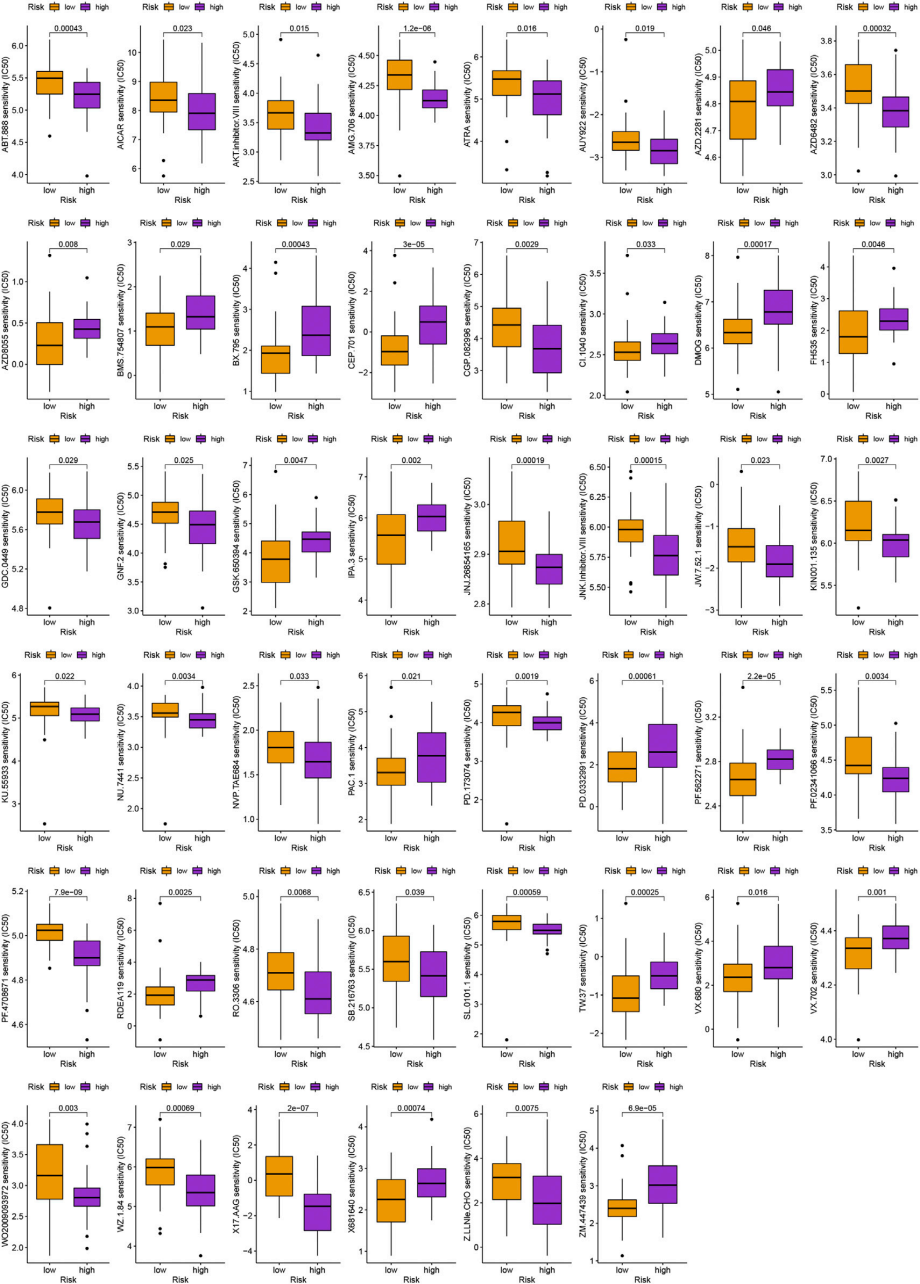
Schematic representation of novel potential agents for uveal melanoma.

In addition to immunotherapy, we are interested in finding innovative targeted therapies and traditional chemotherapeutic agents for patients in different groups. Lastly, we searched for various chemotherapeutic medications and innovative targeted agents for patients in diverse subgroups, which contributed in the creation of customized therapy regimens for unique patients (*p* < 0.05; Figures 6, 7).

## 4 Discussion

The capacity of UM to metastasize exhibits notable heterogeneity. Up to half of UM patients experience distant metastases, most frequently in the liver (Yang et al., 2018; Bakhoum et al., 2021). The median survival is less than 12 months if metastases have been clinically identified (Chattopadhyay et al., 2016). Although the initial tumor was successfully controlled locally, during the past 40 years, overall survival rates have remained stable (Kaliki and Shields, 2017).

Proton beam radiation therapy, enucleation, and iodine or ruthenium plaque radiotherapy are among options for treating UM (Carvajal et al., 2017). Despite being effective in minimizing recurrence and controlling the original tumor, these therapies have little effect on the likelihood of metastasis (Rossi et al., 2021). Targeted therapy would ideally be efficient against both the original tumor and micrometastases (Wu et al., 2022). Immune checkpoint drugs have shown excellent results in treating metastatic cutaneous melanoma and metastatic conjunctival melanoma (in a small number of cases) (Radivoyevitch et al., 2021). However, efforts to use this strategy in metastatic UM have fallen short (Carvajal et al., 2017). Traditional cancer treatments produce great local tumor control, but 50% of patients experience metastases, which almost always have fatal consequences (Carvajal et al., 2017). Targeted medicines are ineffective in the clinic for somatic driver mutations that affect the MAP-kinase pathway (Kaliki et al., 2015). The G protein alpha subunits GNAQ and GNA11, which are the most common driver mutations in UM, are still untreatable (Souto et al., 2019). There are currently no medications available that target the YAP-TAZ pathway, which is also active in UM, the cancer-suppressor gene BAP1, or the SF3B1 gene, whose mutations increase the likelihood of metastatic disease (Li et al., 2019). In the treatment of UM, immunotherapy is only marginally beneficial; anti-CTLA-4 and anti-PD-1 blocking antibodies did not perform as anticipated, with the exception of a few rare cases. Therefore, finding new treatment targets is urgently needed.

Tumor progression involves various biological processes, including tumor cell migration, epithelial-mesenchymal transition, and RNA methylation and modification events such as m6A, m5C, m1A, and m7G, which have been implicated in both *in vitro* and *in vivo* studies. Modification events are also important prognostic indicators in various malignancies (Bakhoum et al., 2021; Motorin and Helm, 2022). Recent research has shown that BAP1 methylation at a single genomic region is highly correlated with BAP1 mutations, BAP1 genomic copy loss, and protein levels that are related to uveal melanoma metastasis, while BAP1 deletion in the initial cancer is related to the disease (Bakhoum et al., 2021). BAP1 methylation has been identified as a prognostic indicator of uveal melanoma spread. Moreover, post-transcriptional enhancement of HINT2 expression by m6A alteration has been shown to indicate advanced uveal melanoma with a poor prognosis (Jia et al., 2019). Studies have also demonstrated that the prognostic value and possible malignant progression of uveal melanoma are significantly influenced by m6A RNA methylation regulators (Wang et al., 2022). Specifically, Guangying Luo et al. have discovered that m6A methylation controls UM cell proliferation, migration, and invasion by focusing on c-Met (Luo et al., 2020). RBM15B, IGF2BP1, IGF2BP2, YTHDF3, and YTHDF1 are five m6A regulators that have been linked to UM patients’ prognoses. It is interesting to note that RBM15 B was found to be the sole independent predictive factor for UM and that there was a strong correlation between it and the clinicopathologic features of UM (Wang et al., 2022). As some research showed, NSUN2-mediated RNA m5C alteration regulates the migration and proliferation of UM cells (Su et al., 2021). As Jiehua Deng et al. identified, m7G may be able to control both CD8^+^ T cells and regulatory T cells (Treg cells), and they also suggest a connection between m7G and the prognosis of melanoma (Chen et al., 2022; Deng et al., 2022). Exploration of m7G-related lncRNA prognostic signature for predicting the immunological state in melanoma was demonstrated by Rong et al. (2022). Additionally, Guangying Luo et al. discovered that NSUN2-mediated RNA m5C modification controls uveal melanoma cell proliferation and migration and that overexpressing miR-124a in UM cells reduced NSUN2 expression levels (Su et al., 2021). Prognostic model and immunological efficacy of m1A-, m5C-, and m6A-related regulators in cutaneous melanoma were found by Xian Rui Wu et al. as potential biomarkers for melanoma research in the future (Wu X. R. et al., 2021). The research outlined above generally indicates that RNA methylation influences the development and prognosis of UM malignancies.

As was already indicated, the signatures currently being utilized to explore the prognosis of UM patients are inadequate and not sufficiently rich. When clinical results and MRGs in UM and the tumor microenvironment were investigated, it was discovered that the TME is very important in UM (Chen et al., 2022). Researchers used MRGs to analyze immune response signatures and make prognostic predictions. These prognostic models in UM were something we wanted to enhance. As a result, we combined information from genes associated with m6A/m5C/m1A/m7G, created a prognostic score, assessed its predictive value and linkage to the immunological landscape, and performed assessments of immune infiltration and medication sensitivity. Such prognostic characteristics can be used independently to explore the results of UM patients more accurately, providing new opportunities for immunotherapy strategies that target UM in the future. Despite significant advances in multimodal therapy, the benefit for survival remains modest. Our research shows that even among UM patients getting the same treatment under the same conditions, the survival advantages were considerably different depending on numerous prognostic factors, including histological grade, tumor stage, and aberrant gene expression (Geeleher et al., 2014). Screening high-risk patients to ensure they receive proper care is therefore crucial, whereas low-risk patients could benefit from appropriate care to prevent long-term toxicity and morbidity (Luo et al., 2020; Li et al., 2022). Building a predictive profile based on aberrant gene expression is particularly crucial to stratify at-risk UM patients and help doctors optimize therapy and make therapeutic decisions (Jin and Jin, 2020).

The role of MRGs in the emergence of malignancies and innate immunity is still being studied. However, it is yet unclear what role the MRGs in UM play clinically. Cancer stage, molecular subtype, cancer mutation load, histological grade and cancer neoantigen load were some of these markers (Yang et al., 2021). This work focused on identifying MRGs, developing a signature, categorizing the patients into various groups, examining the survival rates for various groups within various groupings, and testing the accuracy. The MRGs continued to be individually predictive of prognosis and responsiveness to immunotherapy even after controlling for significant confounders, suggesting their potential as a guiding biomarker for tailored treatment (Jia et al., 2019; Ferrier and Burnier, 2020; Dai et al., 2021). The tumors with elevated MRG levels had a more pervasive immunosuppressive character (Yang et al., 2018; Wang et al., 2022). Furthermore, cancers with high and low-risk showed various TME cell-infiltration characteristics. According to several studies, the MRG’s prognostic characteristic for UM and immune cell subtype invasion are connected (Oliva et al., 2016; Pan et al., 2020; Zhao et al., 2021). Our findings may therefore advance knowledge of how MRGs influence the development of cancer and the antitumor immune response, with significant implications for improved immunotherapy techniques.

## Data Availability

The original contributions presented in the study are included in the article/Supplementary Material, further inquiries can be directed to the corresponding author.

## Supplementary material

SUPPLEMENTARY TABLE S1 The MRGs.

SUPPLEMENTARY TABLE S2 The 689 DEGs.

SUPPLEMENTARY TABLE S3 The GO enrichment analysis.

SUPPLEMENTARY TABLE S4 The KEGG enrichment analysis.
